# Clinical Profile and Outcomes of Patients With Hypercalcemia in an Indian Tertiary Care Center

**DOI:** 10.7759/cureus.46062

**Published:** 2023-09-27

**Authors:** Taniya Sukhija, Vandana Midha, Naveen Mittal, Eva Kalra, Karan Chouhan, Diljot Singh, FNU Dishant, Parmarth Attri, Manjeet K Goyal, Saurabh Arora

**Affiliations:** 1 Internal Medicine, Dayanand Medical College and Hospital, Ludhiana, IND; 2 Endocrinology, Dayanand Medical College and Hospital, Ludhiana, IND; 3 Medicine and Surgery, Dayanand Medical College and Hospital, Ludhiana, IND; 4 Gastroenterology and Hepatology, Dayanand Medical College and Hospital, Ludhiana, IND

**Keywords:** acute pancreatitis, pth & psychosis, hyperparathyroid-induced hypercalcemia, : acute kidney injury, recurrent nephrolithiasis, malignancy related hypercalcemia, asymptomatic hypercalcemia

## Abstract

Hypercalcemia is a complex medical condition characterized by elevated levels of serum calcium (>10.5 mg/dL) in the bloodstream, often arising from various underlying etiologies. This condition presents a significant clinical challenge due to its diverse clinical manifestations and potential for serious complications. Profiling and understanding hypercalcemia is essential for accurate diagnosis, appropriate management, and improved patient outcomes.

In this study, we delve into the comprehensive profiling of hypercalcemia, encompassing its epidemiology, pathophysiology, clinical presentation, and diagnostic approaches. We explore the multifaceted etiological factors contributing to hypercalcemia, including primary hyperparathyroidism, malignancies, granulomatous disorders, medications, and more. We highlight the intricate interplay between parathyroid hormone, vitamin D, and other regulatory mechanisms that influence calcium homeostasis, shedding light on the underlying molecular pathways.

Furthermore, we discuss the diverse clinical manifestations of hypercalcemia, ranging from asymptomatic cases to severe, life-threatening complications involving the renal, gastrointestinal, cardiovascular, and neuromuscular systems. Accurate diagnosis is pivotal, and we evaluate the array of laboratory tests, imaging modalities, and specialized assays that aid in identifying the root cause of hypercalcemia. We emphasize the importance of a systematic approach to differential diagnosis and the significance of risk stratification to guide clinical decision-making.

The evolving landscape of treatment options for hypercalcemia is also explored, encompassing both acute management and long-term strategies tailored to the underlying etiology. We assess the role of hydration, pharmacological agents, and surgical interventions, underscoring the need for individualized therapeutic plans based on the severity and underlying cause of hypercalcemia.

In conclusion, the profiling of hypercalcemia is a multidimensional endeavor that necessitates a comprehensive understanding of its underlying mechanisms, diverse clinical presentations, and diagnostic intricacies. This study intends to serve as a valuable resource for healthcare professionals, offering insights into the complex terrain of hypercalcemia.

## Introduction

Hypercalcemia is defined as the serum calcium concentration two standard deviations above the mean values. The normal serum calcium ranges from 8.5 mg/dL to 10.5 mg/dL [1]. Hypercalcemia is further classified as mild 10.5 to 11.9 mg/dL (2.5-3 mmol/l), moderate 12 to 13.9 mg/dL (3-3.5 mmol/l), and severe >14 mg/dL (>3.5 mmol/l). Calcium homeostasis, which is primarily controlled by the parathyroid hormone (PTH), calcitonin, and vitamin D, maintains adequate calcium levels in the body. Derangements in this process can result in hypercalcemia or hypocalcemia, which significantly negatively impacts health.

Hypercalcemia can be driven by more than 25 different diseases, a number of drugs, and even dehydration. The majority of individuals with hypercalcemia suffer from primary hyperparathyroidism and various types of cancer [2]. Malignancies like squamous cell carcinoma, renal cell carcinoma, breast cancer, multiple myeloma, rhabdomyosarcoma, lymphoma, etc., can lead to hypercalcemia [3]. Among primary hyperparathyroidism, parathyroid adenoma accounts for most cases [3]. Other causes of hypercalcemia include lung diseases such as sarcoidosis and tuberculosis, kidney failure, thyrotoxicosis, Paget’s disease of the bone, immobilization for a prolonged time, and medications like lithium, thiazides, vitamin D, and calcium supplements [4].

Symptoms of hypercalcemia are evident once the serum levels exceed 12 mg/dL which clinically manifests as ‘bones, stones, abdominal moans and physical groans’ [5]. Renal manifestations include nephrolithiasis, distal renal tubular acidosis, nephrogenic diabetes insipidus, and even renal failure in untreated cases [6]. Gastrointestinal symptoms include nausea, vomiting, anorexia, cholelithiasis, pancreatitis, etc. [6]. Mood instability, cognitive dysfunction, and anxiety are a few of its neurological presentations [5]. Patients may also experience musculoskeletal pain, fractures, and myopathies.

After taking a detailed history, and obtaining serum calcium levels, PTH and Vitamin D levels should be estimated to classify the type of hypercalcemia: PTH Dependent or PTH Independent [7]. This can be followed by other investigations like tissue biopsies, serum angiotensin-converting enzyme (ACE) levels, serum protein electrophoresis, etc., to study the specific cause of hypercalcemia [8]. The management of hypercalcemia is based on the treatment of the underlying cause. Still, the primary step is to provide symptomatic relief to the patient by administration of intravenous (i.v.) fluids and diuretics [9]. The presentation and management of hypercalcemia have varied with time and place. In this study, we aim to examine the clinical-etiological profile of patients presenting to our tertiary care center with hypercalcemia and assessment of short-term outcomes in these patients.

## Materials and methods

This prospective study was conducted on adult patients (age > 18 years) with hypercalcemia (corrected calcium >10.5 mg/dL) diagnosed on two separate occasions at a tertiary healthcare center, from 1st January 2019 to 31st December 2019. A total of 200 patients were enrolled in the study after satisfying the inclusion and exclusion criteria and were followed up for a period of three months. A thorough clinical history (demographics, symptoms, past medical history, personal history, drug use, and family history) and general examination were carried out on each subject. Thereafter, an etiological evaluation was carried out and they were followed up for a period of three months. A provisional diagnosis guided specific investigations, such as serum protein electrophoresis, tissue biopsies and aspiration cytology, parathyroid assays, thyroid profiles, serum ACE and 25 hydroxy Vitamin-D levels. The etiology, clinical presentation, course of treatment, and outcome of each patient with hypercalcemia were assessed. Wherever necessary, appropriate radio-nuclear imagings were performed. Ethical clearance was obtained from the "Research and Ethical Committee, Dayanand Medical College and Hospital, Ludhiana, Punjab, India" (BFUHS/2K19p-TH/10723).

The collected data was analyzed using Statistical Package for the Social Sciences (IBM SPSS Statistics for Windows, Version 20.0., IBM Corp., Armonk, NY, USA). For quantitative data, the mean and SD were determined, whereas, for qualitative variables, proportions and percentages were computed. Using the Student t-test and the ANOVA test, quantitative factors between the study groups were compared. The chi-square test was used to compare categorical data. Statistical significance was defined as a probability value (p-value) less than 0.05.

## Results

A study enrolled 200 patients with confirmed hypercalcemia between January 2019 and December 2019. The patients were aged 18-83, with 18.3% aged 18-40 and 40.2% aged 41-60. The majority were male (51.5%) and had mild hypercalcemia (41%). Gastrointestinal symptoms were the most prevalent, with no significant difference in renal symptoms. Neurological symptoms increased from mild to moderate cases, with generalized symptoms more common in moderate and severe groups. Musculoskeletal symptoms increased with severity, with the most prevalent in severe cases (41.1%). Generalized symptoms were more common in the moderate (66.1%) and severe groups (60.7%) compared to the mild group (46.3%), showing statistical significance (p=0.045). Musculoskeletal symptoms increased with severity, being the most prevalent in severe cases (41.1%), and the difference was statistically significant (p=0.012), as depicted in Table 1.

**Table 1:** Baseline characteristics of patients with hypercalcemia n - number of patients; % - percentage of patients; p<0.05 - significant

Parameters	Sub-groups	Hypercalcemia (at admission)			Total	Chi-square value	p-value
		Mild (n=82)	Moderate (n=62)	Severe (n=56)			
Age group (in years)	18-40 [n (%)]	15 (18.3%)	4 (6.4%)	3 (5.4%)	22	8.238	0.221
	41-60 [n (%)]	33 (40.2%)	28 (45.2%)	26 (46.4%)	87		
	61-80 [n (%)]	31 (37.8%)	28 (45.2%)	26 (46.4%)	85		
	>80 [n (%)]	3 (3.7%)	2 (3.2%)	1 (1.8%)	6		
Gender	Male [n (%)]	38 (46.3%)	36 (58.1%)	29 (51.8%)	103	1.945	0.378
	Female [n (%)]	44 (53.7%)	26 (41.9%)	27 (48.2%)	97		
Symptoms	Gastrointestinal [n (%)]	79 (96.3%)	54 (87.1%)	53 (94.6%)	186	4.957	0.084
	Renal [n (%)]	7 (8.5%)	8 (12.9%)	9 (16.1%)	24	1.858	0.395
	Neurological [n (%)]	23 (28.0%)	29 (46.8%)	18 (32.1%)	70	5.721	0.057
	Generalized [n (%)]	38 (46.3%)	41 (66.1%)	34 (60.7%)	113	6.187	0.045
	Musculoskeletal [n (%)]	15 (18.3%)	16 (25.8%)	23 (41.1%)	54	8.825	0.012

The hospitalization outcomes of patients diagnosed with hypercalcemia, classified based on the various causes of the condition and the different outcomes: discharge, death on arrival (DOR), discharge against medical advice (DAMA), and death are summarized in Table 2. Malignancy was the most common cause of hypercalcemia, accounting for 85 patients (36.2% of discharges, 48.4% of DORs, 72.7% of DAMAs, and 44.4% of deaths). Hyperparathyroidism and drug-induced hypercalcemia were the next most prevalent cause, seen in 45 patients (24.6% of discharges, 22.6% of DORs, 9.1% of DAMAs, and 22.2% of deaths) and 32 patients (18.8% of discharges, 9.7% of DORs, 9.1% of DAMAs, and 11.1% of deaths), respectively. However, there was no significant correlation between the cause of hypercalcemia and the hospitalization outcome.

**Table 2:** Outcome of patients with hypercalcemia in hospital n - number of patients; % - percentage of patients; p<0.05 - statistically significant

Parameters	In Hospital Outcome				Total	Chi-square value	p-value
	Discharge (n=138)	Discharge on request (n=31)	Discharge against medical advice (n=27)	Death (n=9)			
Malignancy [n (%)]	50 (36.2%)	15 (48.4%)	16 (72.7%)	4 (44.4%)	85	2.261	0.589
Hyperparathyroidism [n (%)]	34 (24.6%)	7 (22.6%)	2 (9.1%)	2 (22.2%)	45	2.631	0.452
Drug-induced [n (%)]	26 (18.8%)	3 (9.7%)	2 (9.1%)	1 (11.1%)	32	2.692	0.442
Chronic liver disease [n (%)]	10 (7.2%)	1 (3.2%)	0	1 (11.1%)	12	2.262	0.453
Thyrotoxicosis [n (%)]	5 (3.6%)	0	2 (9.1%)	0	7	3.493	0.322
Granulomatous disease [n (%)]	5 (3.6%)	1 (3.2%)	0	0	6	0.952	0.813
Probable malignancy [n (%)]	2 (1.45%)	3 (9.68%)	0	0	5	7.972	0.047
Others [n (%)]	6 (4.3%)	1 (3.2%)	0	1 (11.1%)	8	5.831	0.757

The relationship between the cause of hypercalcemia and patient outcome at three months follow-up was assessed in 200 patients, out of which 22 died, and 178 survived. Hyperparathyroidism was found in 9.1% of the dead and 24.2% of the survivors (p = 0.173), and drug-induced hypercalcemia was found in 9.1% of the dead and 16.9% of the survivors (p = 0.630). Chronic liver disease (CLD) was found in 4.5% of dead patients and 6.2% of survivors (p = 0.533), and thyrotoxicosis was found in 4.5% of dead and 3.4% of survivors (p = 0.603). At the three-month follow-up, only malignancy showed a significant association (p=0.01) with patient outcome, as depicted in Table 3.

**Table 3:** Outcome of patients with hypercalcemia at 3 months of follow-up n - number of patients; % - percentage of patients; p<0.05 - statistically significant

Parameter	Outcome at 3 months of follow-up		Total	Chi-square value	P-value
	Died (n=22)	Alive (n=178)			
Malignancy [n (%)]	15 (68.2%)	70 (39.30%)	85	6.672	0.01
Hyperparathyroidism [n (%)]	2 (9.1%)	43 (24.2%)	45	2.549	0.173
Drug-induced [n (%)]	2 (9.1%)	30 (16.9%)	32	0.934	0.630
CLD [n (%)]	1 (4.5%)	11 (6.2%)	12	1.257	0.533
Thyrotoxicosis [n (%)]	1 (4.5%)	6 (3.4%)	7	1.012	0.603
Granulomatous disease [n (%)]	0	6 (3.4%)	6	0.634	0.426
Probable Malignancy [n (%)]	0	5 (2.81%)	5	0.634	0.426
Other [n (%)]	1 (4.5%)	7 (3.9%)	8	4.405	0.622

## Discussion

The current study was carried out at Dayanand Medical College and Hospital, Ludhiana over a period of 1 year, i.e., 1st January 2019 to 31st December 2019. Consecutive patients from the emergency department with confirmed hypercalcemia on two occasions were enrolled in our study. Patients were followed up at one month and three months intervals respectively. Out of 200 patients, 87 (43.5%) were in the age group of 41-60 years while 85 (42.5%) were in the age group of 61-80 years. The mean age of the cohort was 58.3 years ± 13.6 years (range 18 to 95 years), which was similar to the study conducted by Kuchay et al. (55.2 years ± 17.9) [10]. There was no significant difference in gender distribution in our study sample (103 males and 97 females), which contrasted with another study from north India by Kuchay et al. who studied 552 patients with hypercalcemia and reported male preponderance (332 males and 230 females) [10]. However, our results were in accordance with Western studies by Catalano et al. [11] and Lindner et al. [12]. Catalano et al. reported 585 hypercalcemia patients, out of which 50.9% were females [11]. Similarly, Lindner et al. didn’t show a significant difference in gender distribution (53% females and 47% males) [12].

The majority of our study patients had mild hypercalcemia (n=82, 41%) followed by moderate (n=72, 21.2%) and severe hypercalcemia (n=56, 28%). Our results were comparable with another Asian study by Lee et al. [13] who also reported the majority of the cases with mild hypercalcemia (n=221, 70.7%) followed by moderate (n=72, 21.2%) and severe hypercalcemia (n=26, 8.1%). Malignancy was identified in the majority of symptoms with severe hypercalcemia (n=31, 55.3%) followed by hyperparathyroidism (n=12, 21.4%) and vitamin D intoxication (n=8, 14.2%). This was consistent with another Indian research by Gupta [14] which found that malignancy was the most prevalent cause of severe hypercalcemia (80.9%) followed by hyperparathyroidism (12.3%). These findings were comparable with studies by Azzabi et al. [15], Frolich [16] and Dent et al. [17]. Hence, over the last three decades, literature suggests malignancy and hyperparathyroidism to be the most common cause of hypercalcemia in-hospital context. Therefore, in an admitted patient with severe hypercalcemia, the initial differential should be malignancy followed by hyperparathyroidism and further workup should be planned based on clinical history and examination.

In our study, there was no significant correlation between gender and the severity of hypercalcemia whereas, in the study conducted by Lee et al. [13], severe hypercalcemia was more common in males (20 vs. 8). Furthermore, the follow-up outcome was found to be quite excellent except in cases of malignancy where the maximum mortality was observed. A total of 22 patients died by the end of three months of the follow-up in our study and most of the patients died of malignancy (n=15, 68.2%). This can be attributed to the fact that most of the patients with malignancy were referred to our tertiary care center at a very late stage.

Two patients (9.1%) died of multiorgan dysfunction secondary to pancreatitis in the hyperparathyroidism group in the hospital. The overall prognosis for hyperparathyroidism was found to be favorable, particularly post-parathyroidectomy. One patient (4.5%) died of chronic liver disease (CLD) in the hospital due to decompensation whereas two (9.1%) died of vitamin D toxication in the hospital. One patient (4.5%) had in-hospital mortality due to multiorgan dysfunction secondary to sepsis. One patient (4.5%) with thyrotoxicosis and CLD, died of aspiration of variceal bleed whereas the exact etiology of mortality could not be ascertained in one (4.5%) of patients post-discharge.

Malignancy and hyperparathyroidism are the two most common etiologies associated with hypercalcemia in hospital settings whereas in outpatients, hyperparathyroidism is seen more commonly. The majority of the patients with hyperparathyroidism are diagnosed in an asymptomatic stage in Western countries whereas in India, most of the patients are diagnosed when symptomatic [18]. This difference can be attributed to increased awareness and routine biochemical screening of serum calcium. Vitamin D intoxication is on the rise due to overcorrection of vitamin D deficiency as well as unnecessary prescription of vitamin D for non-specific pains especially in the elderly [19]. Emphasis on patient education should be given for better outcomes. There is a paucity of data on the clinical profile of hypercalcemia patients in India [20]. There is more scope for research in this field to enhance the outcomes of patients with hypercalcemia.

This study had some limitations: Firstly, it was a single-center study and extrapolation of these results on other populations needs to be confirmed. The study had a relatively small sample size and therefore confounding effect of malignancy on outcome could not be studied.

## Conclusions

In conclusion, the profiling of hypercalcemia is an intricate and multifaceted undertaking that holds paramount importance in clinical practice. Through a comprehensive exploration of its epidemiology, pathophysiology, clinical manifestations, and diagnostic methodologies, we have gained valuable insights into the complexity of this medical condition. Hypercalcemia's diverse etiologies, spanning from primary hyperparathyroidism to malignancies and beyond, highlight the need for a systematic and tailored approach to diagnosis and management. As healthcare professionals, it is crucial to recognize the subtle variations in the presentation of hypercalcemia and employ a meticulous diagnostic strategy to identify its underlying cause. The integration of laboratory tests, imaging studies, and specialized assays enables us to differentiate between benign and malignant forms of hypercalcemia and guides us toward informed clinical decisions.

Malignancy and hyperparathyroidism were the most common causes of hypercalcemia in our hospital setting with vitamin D intoxication as an emerging cause of hypercalcemia. Hypercalcemia has varied manifestations therefore hypercalcemia should be included in routine workup. Furthermore, our examination of the profiling of hypercalcemia underscores the importance of a multidisciplinary approach, where acute interventions and long-term management plans are customized to address both the severity of hypercalcemia and its root cause. In our pursuit to optimize patient outcomes, the profiling of hypercalcemia empowers us to navigate through the complexities of this condition. By staying attuned to the latest research, embracing advancements in diagnostic techniques, and tailoring interventions to each patient's unique circumstances, we can effectively manage hypercalcemia and mitigate its potential complications.

In summary, the comprehensive profiling of hypercalcemia serves as an indispensable compass, guiding clinicians in their quest to unravel its intricate nature and offering patients the best possible avenues for diagnosis, treatment, and ultimately, improved quality of life.
